# Unraveling the tripartite interaction of volatile compounds of *Streptomyces rochei* with grain mold pathogens infecting sorghum

**DOI:** 10.3389/fmicb.2022.923360

**Published:** 2022-07-28

**Authors:** A. Sudha, D. Durgadevi, S. Archana, A. Muthukumar, T. Suthin Raj, S. Nakkeeran, Peter Poczai, Omaima Nasif, Mohammad Javed Ansari, R. Z. Sayyed

**Affiliations:** ^1^Department of Plant Pathology, Tamil Nadu Agricultural University, Coimbatore, India; ^2^Department of Plant Pathology, Faculty of Agriculture, Annamalai University, Chidambaram, India; ^3^Department of Biotechnology, Tamil Nadu Agricultural University, Coimbatore, India; ^4^Finnish Museum of Natural History, University of Helsinki, Helsinki, Finland; ^5^Department of Physiology, College of Medicine, King Khalid University Hospital, King Saud University, Riyadh, Saudi Arabia; ^6^Department of Botany, Hindu College, (Mahatma Jyotiba Phule Rohilkhand University, Bareilly), Moradabad, India; ^7^Department of Microbiology, PSGVP Mandal’s S. I. Patil Arts, G. B. Patel Science, and STKV Sangh Commerce College, Shahada, India

**Keywords:** antifungal, grain mold, interaction, mVOCs, sorghum, *S. rochei*

## Abstract

Sorghum is a major grain crop used in traditional meals and health drinks, and as an efficient fuel. However, its productivity, value, germination, and usability are affected by grain mold, which is a severe problem in sorghum production systems, which reduces the yield of harvested grains for consumer use. The organic approach to the management of the disease is essential and will increase consumer demand. Bioactive molecules like mVOC (volatile organic compound) identification are used to unravel the molecules responsible for antifungal activity. The *Streptomyces rochei* strain (ASH) has been reported to be a potential antagonist to many pathogens, with high levels of VOCs. The present study aimed to study the inhibitory effect of *S. rochei* on sorghum grain mold pathogens using a dual culture technique and *via* the production of microbial volatile organic compounds (mVOCs). mVOCs inhibited the mycelial growth of *Fusarium moniliforme* by 63.75 and *Curvularia lunata* by 68.52%. mVOCs suppressed mycelial growth and inhibited the production of spores by altering the structure of mycelia in tripartite plate assay. About 45 mVOCs were profiled when *Streptomyces rochei* interacted with these two pathogens. In the present study, several compounds were upregulated or downregulated by *S. rochei*, including 2-methyl-1-butanol, methanoazulene, and cedrene. *S. rochei* emitted novel terpenoid compounds with peak areas, such as myrcene (1.14%), cymene (6.41%), and ç-terpinene (7.32%) upon interaction with *F. moniliforme* and *C. lunata*. The peak area of some of the compounds, including furan 2-methyl (0.70%), benzene (1.84%), 1-butanol, 2-methyl-(8.25%), and myrcene (1.12)%, was increased during tripartite interaction with *F. moniliforme* and *C. lunata*, which resulted in furan 2-methyl (6.60%), benzene (4.43%), butanol, 2-methyl (18.67%), and myrcene (1.14%). These metabolites were implicated in the sesquiterpenoid and alkane biosynthetic pathways and the oxalic acid degradation pathway. The present study shows how *S. rochei* exhibits hyperparasitism, competition, and antibiosis *via* mVOCs. In addition to their antimicrobial functions, these metabolites could also enhance plant growth.

## Introduction

Sorghum is a staple food for people in the semi-arid tropics. Sorghum is mainly cultivated during India’s Kharif and Rabi seasons (Manzar et al., 2021). Several diseases were reported in sorghum crops in various parts of Tamil Nadu, with the majority of them being seed-borne. Grain mold, the most widespread and significant sorghum disease globally, is a significant constraint on sorghum productivity. Grain mold progress is especially vigorous in short-term hybrid cultivars and varieties grown in temperate and sultry conditions during the rainy season. Several grain mold fungi, such as *Curvularia lunata*, *Fusarium moniliforme*, *Alternaria alternata*, *Macrophomina phaseolina*, *Rhizopus stolonifer*, *Phoma sorghina*, *Drechslera rostrata*, and *Aspergillus* spp., can infect sorghum (Sajjan et al., 2014). *Fusarium moniliforme* and *Curvularia lunata* are counteracted due to carbohydrate absorption, the budding of the kernel, and the triggering of seeds, which decreases their size and weight without visible fungal development (Prom et al., 2016; Das et al., 2020). The grain mold fungi cause grain deterioration, reduced seed weight, poor germination, loss of viability, and death of seedlings (Cuevas et al., 2019; Das et al., 2020). Hence, an economic and safe method of mold control would greatly help the use of sorghum grains both for food and feed. Several biological agents, including *Trichoderma hamatum*, *T. koningii*, *Bacillus subtilis*, *Pseudomonas fluorescens*, and *Streptomyces* spp., have demonstrated promising results in the laboratory and in the field (Kashyap et al., 2021; Manzar et al., 2021). Protecting crops against grain mold through pesticides is possible, but unsystematic application creates a stronger reaction that threatens the environment through residual effects.

Plant growth-promoting rhizobacteria increase the use of phosphate solubilization (Sharma et al., 2016), improve nutrient availability in plants (Hamid et al., 2021; Sarkar et al., 2021), and biosynthesize metal chelators (Nithyapriya et al., 2021). Most PGPR have been used to control phytopathogens (Khan et al., 2021; Sukmawati et al., 2021) and ease abiotic stress in plants (Kashyap et al., 2017, 2021; Olanrewaju et al., 2017; Zhao et al., 2019; Sagar et al., 2020, 2022a,b; Kusale et al., 2021a,b). Actinobacteria, such as *Streptomyces* spp., have been used to manage phytopathogens through biological control (Katarzyna et al., 2018; Kalam et al., 2020). The mode of action of *Streptomyces* sp. is the production of cellulolytic enzymes, such as cellulases, chitinases, amylases, and glucanases, by pathogenic fungi during the interaction. Light and scanning electron micrographs have been used to determine the effects of *Streptomyces* sp.-parasitizing phytopathogenic fungus (Miyada et al., 2017; Kong et al., 2019). The interface between the biocontrol agent and pathogens may upregulate or downregulate certain semiochemicals. Although there are numerous studies on microbe-specific mVOCs, the full range of their interactions with infections and Actinobacteria is yet to be explored (Sharma et al., 2020). Furthermore, *Streptomyces* spp. is well-known for the secretion of specific organic compounds with pharmaceutical applications and producing natural bioactive secondary metabolites (70–80%) in large quantities (Salwan and Sharma, 2020).

Hence, our study shows that *S. rochei* confers biocontrol potential by releasing an array of compounds. Identifying the mVOCs involved in antagonists might lead to more efficient strategies for grain mold management. The present study demonstrates that organic compounds from *S. rochei* are the major key compounds in managing sorghum grain mold pathogens. Using GC-MS-TD techniques, *S. rochei* is shown to excrete many volatile chemicals in the present study. Laboratory studies were carried out to determine the antagonistic effects of volatile amalgamates in *S. rochei* against *Fusarium moniliforme* and *Curvularia lunata.* We analyzed the main components using the PCA to understand their mutual relationships among the treatments. The PCAs with eigenvalues more than 1 were considered method; the observations vary, and subgroups were calculated.

## Materials and methods

### Microorganisms: Isolation and identification

The biocontrol strain *Streptomyces rochei* (NCBI Accession No: MT122809) was obtained from Culture Collection Centre, Department of Plant Pathology, and was isolated from the rhizosphere of sun hemp in the TNAU Campus, Coimbatore, India. The starch casein agar medium was used for culturing *Streptomyces rochei*; 25 μg/ml of nystatin was added to the medium to reduce fungal contamination. After a week, the colony of *S. rochei* was selected based on their morphological characteristics, sporulation was induced from a solid medium using International Streptomyces Project Medium ISP-4, and ISP-2 was used for fermenting the culture. This isolate was identified by the 16S rRNA genes and the genetic relatedness of *S. rochei* was inferred using phylogenetic relatedness. CLUSTAL_W was used to align the sequences. Phylogenetic trees were built to assure the reliability and stability of phylogenetic connections using the strains available in NCBI GenBank database strains.

Sorghum grain mold causative organisms, namely, *Fusarium moniliforme* and *Curvularia lunata*, were isolated from the infected sorghum grains. The infected sorghum grain tissue portions were cut into small pieces of 1.0 cm^2^ and surface-sterilized with sodium hypochlorite 0.1% for 30 s. Then, they were rinsed with sterilized water three times and dried using tissue paper. The sterilized infected plant tissue sections were then placed on culture plates with 20 ml of PDA medium and conditioned at 25°C for further development. A single hyphal tip technique was used to purify and maintain the cultures. The grain mold infections were initially identified based on phenotypic characteristics and then validated using specific primers. Kusai et al. (2016) described the primers P1 and P2 with their nucleotide sequence of *Clg2p* Ras protein gene for *C. lunata* identification at 870 bp and the translational elongation factor (TEF-1) gene of *F. moniliforme* at 420 bp. The results were determined after amplification using a Gel Doc XR system.

### Screening of antifungal activity of *S. rochei* against *F. moniliforme* and *C. lunata*

The *S. rochei* strain (ASH) was evaluated for antifungal activity against the pathogens by a dual plate-assay technique on PDA in Petri dishes (Djellel and Larous, 2018). For each fungal strain, 5-day-old actively growing mycelium of 9 mm diameter was placed on the opposite side, and *S. rochei* was streaked on the other edge of the Petri plate. These plates were incubated at 28 ± 2°C for 6 days or until the test pathogen covered 9 mm in control. The suppression of test pathogen fungal mycelium was measured. The inhibition zone was measured by the mycelial growth compared to control.


$$\text{Growth}\text{inhibition}=\frac{1-\mathrm{dr}}{\text{dc}}\times 100$$

where dr is the diameter of mycelia in the dual plate and dc is the diameter of mycelia in the untreated plate.

The interactive area of about 1 cm^2^ (zone of inhibition) between the pathogen and *S. rochei* was cut down and examined under the dissection microscope at 400× magnification.

### Tripartite plate assay for inhibition induced by volatile compounds

The growth inhibition of pathogens mediated *via* volatile compounds was studied using tripartite plate assay or divided plate assay. A plate containing potato dextrose agar was radially divided into three parts. An 8-mm agar plug of *F. moniliforme* was placed on one part of the tripartite plate. *C. lunata* was streaked on the second part and sealed using parafilm. The third part was left empty. The setup was incubated at 28 ± 2°C. The control plate consisted of only the pathogen and not the antagonist. The radial growth of pathogen mycelia was measured at 24-h intervals until the control plate was fully covered with growth. In another set of plates along with the pathogens, antagonist was filled in the third part of the tripartite plate to absorb the volatile compounds released by *S. rochei.* The growth of hyphae was measured at regular intervals, and the percentage of mycelia inhibition of the pathogen was determined by comparing it to the control plate.


Percentageofinhibition=[1-(G-eG)a]×100

where G_*e*_ is the mycelial development of the pathogen in the presence of *S. rochei* and G_*a*_ is mycelial development of the pathogen in the absence of *S. rochei*.

### Extraction of the antifungal metabolite

*Streptomyces rochei* was inoculated in a 250-ml conical flask containing ISP-1 medium (casein enzymic hydrolyzate: 5 g; yeast extract: 3 g; distilled water: 1 liter) with a 2-cm^2^ cell plug from a new slant. Then it was kept in a rotary shaker at 150 rpm for 7 days at 28 ± 2°C. The broth was centrifuged for 20 min at 4°C at 10,000 rpm. The resultant solution was filtered and stored after adjusting the pH to 2.0 using 1N HCl. Ethyl acetate was added at equal volume as a solvent, and the mixture was kept for overnight incubation in a shaker at 150 rpm; then, it was extracted twice with ethyl acetate. Antifungal compounds in a solvent were pooled and concentrated through evaporation in a vacuum flash evaporator at 80 rpm at 55°C until condensed. The concentrated liquid was filtered through a 0.22-μm (disposable sterile–Whatman No.) aseptic microporous filter membrane. The filtered metabolite was diluted with methanol (HPLC grade) at various concentrations for further experiments.

### Screening of antifungal metabolite against fungal pathogens

The diluted metabolite from the antagonist was further tested for its antagonistic property against the two fungal pathogens isolated from sorghum. The antagonistic activity of the isolates was tested using an agar well diffusion assay. Then 100 μl of crude metabolite was poured into PDA wells. In each plate, the test pathogen mycelia were inoculated with a 9-mm-diameter agar plug in the center of the Petri dish. The inhibition percentage was calculated after 7 days.

### Microbial volatile organic compound collection with tenax columns and GC-MS-TD analysis

The purge and trap method was used for analyzing volatile compounds. The headspace mVOCs were absorbed by Tenax-coated columns (PerkinElmer cat #HO244966) made of stainless steel. Totally, four sets, including *S. rochei* (1 ml of 72-h-old spore suspension) with grain mold pathogens (8 mm 5-day-old mycelia disk), were inoculated in potato broth (PD broth): *S. rochei* alone, *F*. *moniliforme* or *C. lunata* alone, and both upon tripartite interaction (*S. rochei* vs. *F. moniliforme* and *C. lunata*). Uninoculated PD broth was used as a pessimistic control for the headspace samples. To avoid the dispersal of volatile compounds from the columns, they were sealed with parafilm after the sterilized rubber cork was inserted. The experiment was repeated three times; then, the mycelium was allowed to grow at 28 ± 2°C for 7 days.

The biochemicals produced in the samples were identified using GC-MS, thermal desorber (TD). The resultant mVOCs were compared with NIST 14 standards (Mass Spectral Library). Volatile compounds with a mass spectral resemblance of more than 90% to those in the National Institute of Standards and Technology (NIST) library were categorized as putative active compounds.

### Principal component analysis and heat map

The NIST database extracted the metabolite signals using the MALDIquant wrap up in the RStudio interface. Data normalization was performed using principal component analysis (PCA).

### Seed bacterization and plant growth promotion

Sorghum seeds (cv. CO30) were surface-sterilized for 30 s with 2% sodium hypochlorite, and 72-h-old *S. rochei* was inoculated in a conical flask containing appropriate broth. The cell suspension, comprising 50 g of seeds, was treated with 3 × 10^6^ colony-forming units/ml for 2 h and dried under shade. The seedling vigor index was used to test the plant growth-boosting capabilities of the isolates. Overall, 15 seeds were placed on presoaked germination paper. The same germination paper sheet was lightly folded over the seeds to keep them in place. The polyester layer was then folded up with the seeds and placed inside the humidified incubator for 14 days. Then, three replications were maintained for each treatment. Germination percentage and shoot and root measurements were recorded for each seedling at 7 days after incubation. The vigor index was calculated using the formula of Ali et al. (2021) and expressed as the percentage of germination multiplied by the seedling length.

### Microbial volatile organic compound plate bioassay and plant growth stability

About 10 seeds were placed on the MS agar in a Petri plate, and the potential antagonist *S. rochei* was cultured on 90-mm SCA plates. Both sets were placed and subjected to 24-h light and dark cycles, followed by 12 h under 55 W light in a plant growth chamber at 26 ± 1°C and 60–70% relative humidity. The sorghum seeds CO30 were disinfected for 10 min with sodium hypochlorite 0.1 or 70% ethanol and rinsed three times with deionized water. These sorghum seedlings were periodically monitored for 14 days after seeding to examine the effect of mVOC on growth rates. The length, weight, and quantity of shoot and root were measured.

### Evaluation for *S. rochei* against grain mold pathogens under glasshouse conditions

The pot culture experiments were conducted with a susceptible sorghum cultivar (CO30) at a PL480 glass house, TNAU, Coimbatore district, Tamil Nadu, India. Surface-sterilized seeds were soaked in *S. rochei* spore suspension (at 10^7^ cfu/ml; grown in SCB) and in sterilized water for control for 1 h. The treated seeds were sown immediately in the portrays at 3 cm depth. After germination, the plants were dipped in *S. rochei* spore suspension (at 10^7^ cfu/ml) for a period of 30 min and planted in pots (3/pot). Booster doses of *S. rochei* (5 ml per seedling, 10^7^ cfu/ml) were applied as foliar spray at 50% flowering and 100% flowering stage. The grain mold pathogen (spore suspension of *F. moniliforme* and *C. lunata*) was inoculated as foliar spray during the flowering stage, which served as positive control and healthy control were maintained as negative control. After spray, the panicles were immediately covered with selfing bags and removed after 2–3 days of incubation period. After 24 h and 20 days of foliar spray, around 100 grains (20 grains/panicle) of sorghum from each treatment were harvested from five randomly selected panicles. Furthermore, percentage grain mold severity rating in the field (PGMSR) and percentage mold threshed rating (PMTGR) in the laboratory were evaluated on the harvest at the physiological maturity stage of the crop using a 1–9 rating scale, where 91/4 76–100% molded grains (extremely susceptible), 81/4 51–75% molded grains (highly susceptible), 71/4 41–50% molded grains (susceptible), 61/4 31–40% molded grains (susceptible), 51/4 21–30% molded grains (moderately resistant), 41/4 11–20% molded grains (moderately resistant), 31/4 6–10% molded grains (resistant), 21/4 1–5% molded grains (resistant), and 11/4 no mold (highly resistant) (Thakur et al., 2007). The percent disease index was calculated using the following formula.


$$\begin{array}{c} \text{Percent}\mathrm{disease}\mathrm{index}\\ =\frac{\text{Sum}\text{of}\text{individual}\text{ratings}}{\text{No}\text{of}\text{observation}\times \mathrm{Maximum}\mathrm{grade}} \end{array}$$

### Statistical analysis

All the experiments were performed in triplicate, and the mean value was statistically analyzed using STAR 2.0.1. The *F*-value (*P* = 0.05) was used to determine the significant amount of treatment. The mean and standard deviation of plant growth metrics were obtained, and additional comparisons were made using DMRT at P0.05 (XLSTAT).

## Results

### Molecular confirmation of sorghum grain mold pathogens

The potential biocontrol strain *S. rochei* ASH that showed the highest inhibitory activity and plant growth promoting abilities was used in this study. The P1 and P2 primers were used for amplification and yielded an amplicon of 870 bp for *C. lunata* (Supplementary Figure 1). No amplification was observed when a fungal isolate from a different species was utilized as a negative control (*Fusarium moniliforme*). These findings show that *Curvularia* sp. discovered was *C. lunata*. For *Fusarium* sp., TEF-1 gene synthesis using Fu3f and Fu3r primers revealed a band of 420 bp (Supplementary Figure 1). The TEF-1 gene fragment was sequenced. BLAST search in NCBI revealed that it originated from *Fusarium* sp. because *F. moniliforme* had 99% similarity with related sequences in the GenBank database (Supplementary Figure 1). The phylogenetic relatedness using 16S rRNA sequences was used for the identification of *S. rochei.* The results revealed that the *S. rochei* strain ASH showed 95% similarity with *Streptomyces* sp. strain of Indonesia (Supplementary Figure 2).

### Screening of antifungal activity of *S. rochei* against *F. moniliforme* and *C. lunata*

**FIGURE 1:**
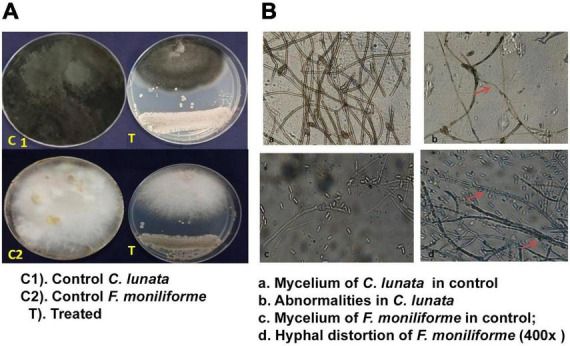
Antagonistic effect of *S. rochei* against grain mold pathogens of Sorghum. **(A)** Dual culture technique. **(B)** Interaction of pathogen with *S. rochei*. Red arrows indicate effect of *S. rochei* on mycelia of pathogen.

The Actinobacteria, *S. rochei*, suppressed the mycelia of both the pathogens (*F. moniliforme* and *C. lunata*) (Figure 1). The direct interaction of the antagonist with the pathogen resulted in changes in the mycelia pattern. The antagonist inhibited the mycelia of *F. moniliforme* by 63.75, and *C. lunata* by 68.52%. The highest inhibition ranged from 65.33 to 68.88%, recorded after 4 days of incubation (Figure 1A). The antagonist, *S. rochei*, caused extensive hyphal thinning and a less dense hyphal network than the control. Light microscopy of the fungal mycelia revealed distortions, damage, and shrinkage of the *C. lunata* conidia in the treated plates. In *F. moniliforme*, the hyphae were parasitized by antagonist spores (Figure 1B). In the control plate, no such changes were detected.

### Tripartite plate assay for inhibition induced by volatile compounds

**FIGURE 2:**
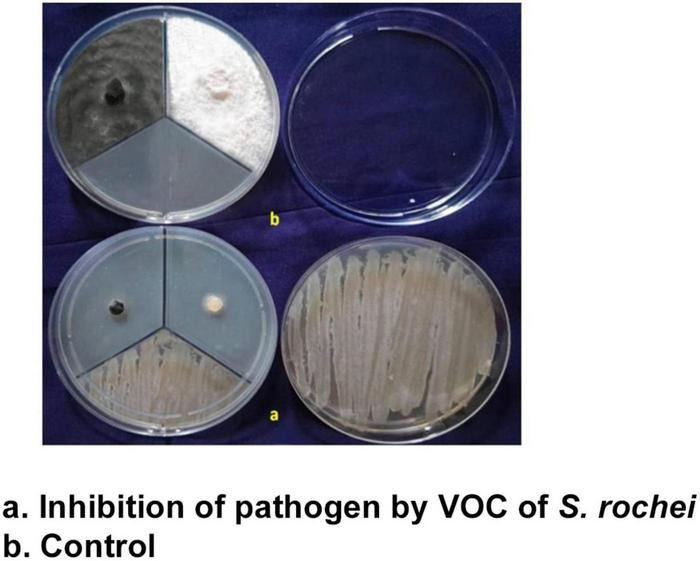
Volatiles of *S. rochei* against sorghum grain mold pathogens (tripartite plate assay).

The volatile compounds emitted by the isolate *S. rochei* were tested against the two pathogens using a tripartite plate assay. This demonstrated that volatile compounds adversely affected the mycelium growth at 9.85 for *F. moniliforme* and 9.02% for *C. lunata* after 3 days. Mycelial growth was reduced by 88 and 89% (82.36 and 88.25%) compared to control (82.36 and 88.25%). The inhibition percentage for both pathogens increased above 90% on day 4 compared to the control (90%) (Figure 2). The effectiveness of the volatile and non-volatile compounds on the mycelial growth of the test pathogens proved the suppressing ability of the antagonist under *in vitro* conditions.

### Validation of antifungal metabolites against the pathogens

**FIGURE 3:**
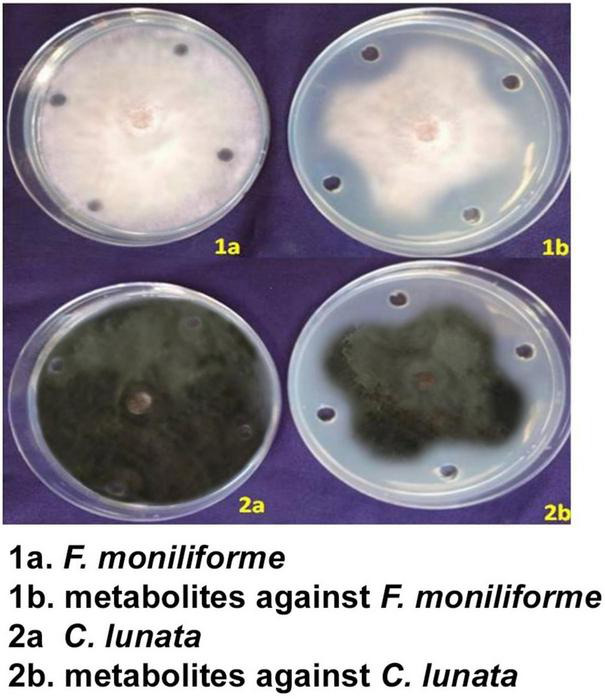
Efficacy of crude metabolites of *S. rochei* against sorghum grain mold pathogens (agar well diffusion assay).

The antimicrobial metabolite extracts (dilution of 100 μl) were poured in three replications along with the control. In the agar well diffusion method, the metabolite of *S. rochei* showed good antifungal activity against the test pathogens. The crude extract inhibited *F. moniliforme* mycelia by 59.65 and *C. lunata* mycelia by 61.54%. In control, the diameter of the radial mycelial growth was 88.54 and 88.61% for these pathogens, respectively. Using 100 μl of the extract showed complete inhibition on the 5th day in the *S. rochei* metabolite compared to the control (Figure 3).

### Volatilome profile associated with *S. rochei*, *F. moniliforme*, *C. lunata*, and their interaction

*Streptomyces rochei* emitted 14 volatile compounds individually with high peak area abundance. The compounds with peak area abundance were furan 2-methyl (0.70), 5-aminovaleric acid (1.19), methyl-D-glucamine (1.18), cyclopentanetriol (9.43), 1-butanol, 2-methyl (8.25), furan 3-methyl (7.92), hydroxyl pyridine (0.60), benzene (1.80), methyl isovalerate (6.00), butanoic acid, 3-methyl-ethyl ester (6.27), ethyl tiglate (2.70), butanoic acid butyl ester (3.79), alpha-phellandrene (2.20), and methyl undecanol (1.64).

During tri-tropic interaction with pathogens (*Fusarium* and *Curvularia*), only three compounds were upregulated: furan 2-methyl (6.60), 1-butanol, 2-methyl (18.67), and benzene (4.43), while cyclopentanetriol and alpha phellandrene were downregulated, and the respective peak area percentage was 2.11 and 0.95. All other compounds were nullified during the interaction. Hence, the furan 2-methyl, 1-butanol, 2-methyl, and benzene were reported as novel antagonistic compounds for the degradation of pathogenic hyphae and distortion of spores.

*Fusarium moniliforme* exerts volatile organic compounds with peak area such as neopentyl alcohol (19.78), tributylamine (8.88), acetic acid (7.91), succinaldehyde (0.48), aminocyanoacetic acid (7.01), oxalic acid (3.17), allantoic acid (2.62), pyruvaldehyde (6.14), formic acid butyl ester (1.15), and homocysteine (3.55). The highest peak area abundance compounds were detected only during *F. moniliforme* alone. During tripartite interaction, all these virulent compounds were nullified.

**FIGURE 4:**
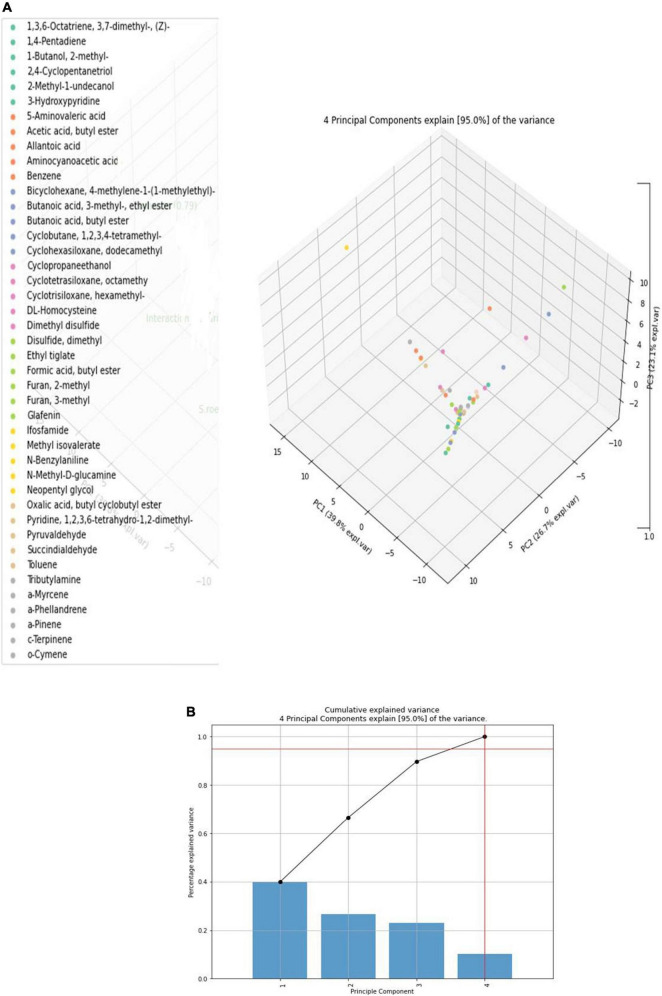
**(A)** Classes of volatile compounds obtained from axenic and co-cultivation. **(B)** Scree plot for volatile compounds obtained in GC-MS-TD in axenic and co-culture.

The results showed that *S. rochei* explored several compounds with peak areas, such as myrcene (1.14%), cymene (6.41), 1,2,4-cyclopentanetriol (6.42), 1-butanol, 2-methyl- (6.47), furan, 3-methyl- (6.71), and ç-terpinene (7.32). *F. moniliforme* alone released compounds such as propanoic acid, (5.37), allantoic acid (5.71), and pyruvaldehyde (6.0). The compounds released by the fungus *C. lunata* were 3-cyclopentene-1,2-diol (4.68), 5-aminovaleric acid (5.19), benzene (5.40), 1-butanol, 2-methyl- (6.29), disulfide, dimethyl (6.70), ethyl ester (7.24), cyclotrisiloxane (7.31), dimethyl trisulfide (9.46), and α-phellandrene (9.88). Upon interaction with *F. moniliforme* and *C. lunata*, for some compounds, including furan 2-methyl (0.70), benzene (1.84), 1-butanol, 2-methyl- (8.25), and myrcene (1.12), the peak area percentage increased during the interaction (6.60, 4.43, 18.67, and 1.14). However, the peak area percentage of 9.43, corresponding to 1,2,4-cyclopentanetriol, decreased during the interaction of *F. moniliforme* and *C. lunata* with *S. rochei* (Figure 4A).

Totally, 45 volatile compounds were produced by *S. rochei* interaction with sorghum grain mold pathogens (Table 1). Our primary objective was to determine how competitive relationships between *F. moniliforme*, *C. lunata*, and *S. rochei* affected the volatile profiles. PCA was used on the volatile biomarkers of axenic and co-cultivated samples, taking into consideration four groupings of samples made up of (1) *F. moniliforme*, (2) *C. lunata*, (3) *S. rochei*, and (4) interaction of *F. moniliforme* + *C. lunata with S. rochei.* Alkanes, alkenes, alcohols, esters, ketones, sulfur, and terpenoid compounds were among the emitted VOCs. 1-Butanol, furan 2, and furan 3-methyl, 1,2,4-cyclopentanetriol, benzene, methyl isovalerate, cyclopentanone, butanoic acid 3 methyl ester, and methyl undecanol were among the compounds most frequently produced by *Streptomyces*. PCA would explain up to 95% of the variation in the dependent variable in terms of volatile profiles, depending on the instances (equal to the total of the scatter plot values for both axis/principal components PC1 and PC2).

**TABLE 1:** Compounds obtained during axenic and co-culture by GC-MS analysis.

Compounds	*F. moniliforme*	*C. lunata*	*S. rochei*	Interaction (*F. moniliforme* + *C. lunata* + *S. rochei*)
Furan, 2-methyl	0	0	0.7	6.6
5-aminovaleric acid	5.2	8.29	1.19	0
N-methyl-D-glucamine	0	0	1.18	0
2,4-cyclopentanetriol	0	0	9.43	2.11
1-butanol, 2-methyl-	0	0	8.25	18.67
Furan, 3-methyl	0	0	7.92	0
3-hydroxypyridine	0	0	0.6	0
Benzene	0	2.19	1.84	4.43
Methyl isovalerate	0	0	6	0
Butanoic acid, 3- methyl-, ethyl ester	0	0	6.27	0
Ethyl tiglate	0	0	2.7	0
Butanoic acid, butyl ester	0	0	3.79	0
α-phellandrene	2.83	1.21	2.2	0.95
2-methyl-1-undecanol	0	0	1.64	0
Cyclopropaneethanol	0	9.02	0	2.11
Benzene	0	2.19	0	4.43
Disulfide, dimethyl	0	13.79	0	7.44
Cyclotrisiloxane, hexamethyl-	5.57	1.9	0	1.87
N-benzylaniline	0	0	0	1.21
Cyclotetrasiloxane, octamethyl	0.79	0	0	0.58
a-myrcene	0	1.12	0	1.14
o-cymene	0	0	0	6.41
c-terpinene	0	0	0	7.32
Cyclohexasiloxane, dodecamethyl	0	0	0	2.23
1,4-Pentadiene	0.56	1.7	0	0
Cyclobutane, 1,2,3,4-tetramethyl-	0	5.95	0	0
Pyridine, 1,2,3,6-tetrahydro-1,2-dimethyl-	0	0.58	0	0
Toluene	0	2.41	0	0
α-Pinene	0	0.47	0	0
Glafenine	0	1.77	0	0
Dimethyl disulfide	0	3.44	0	0
a-myrcene	0	1.12	0	0
Bicyclohexane, 4-methylene-1-(1-methylethyl)-	0	12.29	0	0
1,3,6-octatriene, 3,7- dimethyl-, (Z)-	0	3.86	0	0
Succindialdehyde	0.48	0	0	0
Aminocyanoacetic acid	7.01	0	0	0
Allantoic acid	2.62	0	0	0
Oxalic acid, butyl cyclobutyl ester	3.17	0	0	0
Ifosfamide	6.99	0	0	0
Tributylamine	8.88	0	0	0
Pyruvaldehyde	6.14	0	0	0
Formic acid, butyl ester	1.5	0	0	0
Neopentyl glycol	19.78	0	0	0
DL-Homocysteine	3.55	0	0	0
Acetic acid, butyl ester	7.91	0	0	0

**FIGURE 5:**
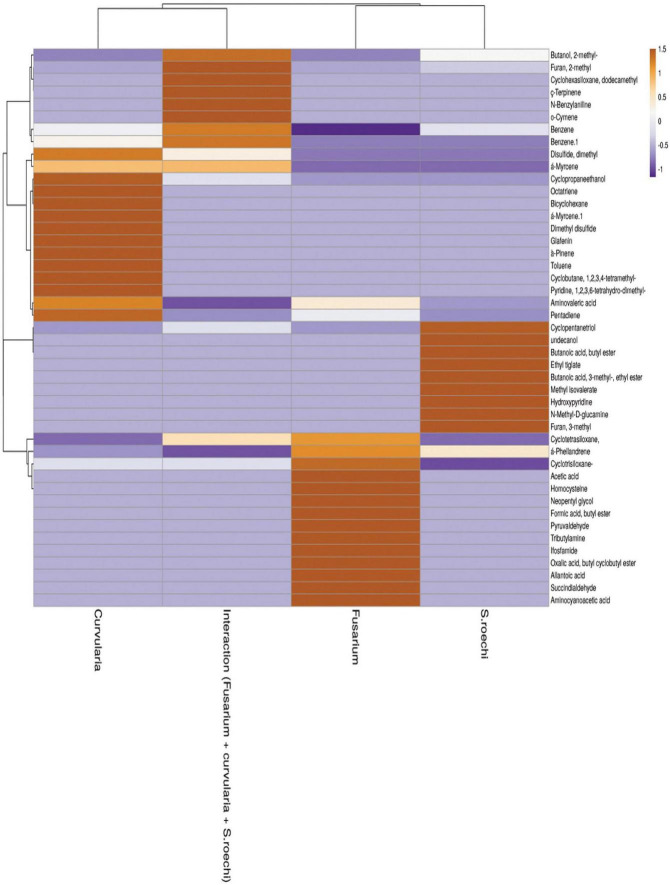
Heat map obtained for upregulated and downregulated volatile compounds in axenic and co-culture.

Principal component scores in the scatter plot and the loading graphs are given in Figure 4A. Principal component 1 (PC1) and principal component 2 (PC2) accounted for 95% of the variance and were statistically significant in representing all variables (Figure 4B). PC1 was related to volatile chemicals such as 2-methyl-1-butanol and methanoazulene; cedrene accounted for 39.8% of the variance. PC2 was also substantially linked with volatile chemicals, accounting for 26.7% of the overall variance, 1 butanol 2-methyl, and 1,2,4 cyclopentanetriol. The treatment with the interaction of *S. rochei* with *F. moniliforme* and *C*. *lunata* aggregated into the same cluster. The association between the values and volatile loadings was on the optimistic side for PC1. Small amounts of N-benzylaniline, cyclohexasiloxane, dodecamethyl, cyclotetrasiloxane, octamethyl, and α-phellandrene were constantly released during the interaction. Figure 5 reports the heat map obtained, analyzing 43 volatile compounds that differentially accumulated among *S. rochei* alone and in tripartite interaction with sorghum grain mold pathogens. Overall, we found that tripartite interaction produced a higher number of VOCs at lower concentrations (purple), while lower numbers of chemical compounds (cyclopentanetriol, undecanol, butanoic acid, butyl ester, ethyl tiglate, 3- methyl-, ethyl ester, hydroxypyridine, N-methyl, and D-glucamine) at higher concentrations (brown) were observed in *S. rochei*, *F. moniliforme*, and *C*. *lunata* alone.

### Seed germination by volatile compounds

**FIGURE 6:**
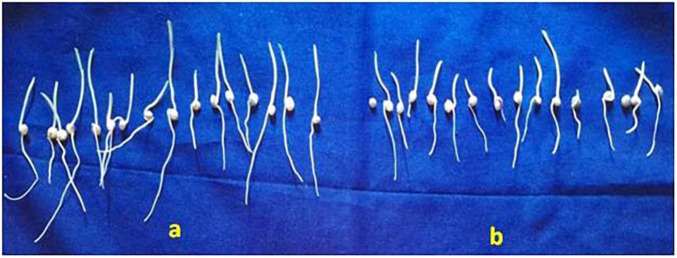
Efficacy of *S. rochei* on plant growth promotion by the roll-towel method. **(A)** Treated with culture filtrate of *S. rochei*; **(B)** Treated with sterile water (control) on the 7th day of germination.

In the roll towel method, *S. rochei*-treated seeds stimulated root and shoot length a day before germination. The mean root length (7.63 cm) and shoot lengths (3.07 cm) were significantly increased in *S. rochei*-treated seeds. Untreated seeds recorded a root length of 1.99 cm, whereas shoot length was 1.67 cm (Figure 6a). The vigor index was 878 in the *S. rochei*-treated seeds and control; this was reduced to 602. The same pattern was seen in the fresh mass of the seedlings (15 No.), which was recorded at 3.72 g in treated and 0.89 g in control after 14 days. This observation supported a 47% increase in the dry plant biomass of sorghum seedlings germinated from treated antagonist seeds (Figure 6b).

**FIGURE 7:**
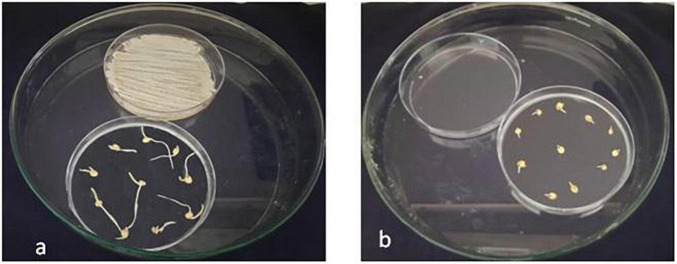
Growth attributes of sorghum seedlings exposed to VOC of *S. rochei*. **(A)** Seeds exposed to volatiles of *S. rochei*; **(B)** Seeds unexposed to volatiles of *S. rochei* (control).

The volatiles released from the antagonist activates the sorghum seedling growth. An *in vitro* bioassay (sorghum CO30) was carried out, in which 10 seeds in each plate were exposed and 10 non-exposed to VOCs. After 14-day incubation, the shoot length was higher in mVOC-exposed plates after 5 days and gradually increased to 49.6% over control. But the root length was reduced in VOC-exposed plants by 50% compared to control. However, an increase in lateral roots of 50% has been recorded in VOC-exposed plants. Furthermore, biomass increased by 52% in VOC-exposed seedlings (Figure 7a) compared to controls (Figure 7b).

### Evaluation for *S. rochei* against grain mold pathogens under glasshouse conditions

The seed treatment with *S. rochei*, followed by seedling dip and foliar spraying, recorded least disease severity (17.00) compared to the control (97.00). The combined application of seed treatment, seedling dip, and soil application also increased the seed weight (3.43) and panicle weight (67.82 g), whereas untreated plants recorded less seed weight (1.90) (Table 2).

**TABLE 2:** Evaluation for *S. rochei* against grain mold pathogens under glasshouse conditions.

Treatments	Grain mold rating (1–9 scale)		Disease severity		Yield parameters	
	Field grade	Thresh grade	Field	Thresh	100 seed weight (g)	Weight of the individual panicle (g)
Seed treatment	4.89	4.72	54.33^e^	52.44^f^	2.69	50.24
Seedling dipping	4.01	3.89	44.55^d^	43.22^e^	2.70	57.41
Foliar spraying	3.78	3.45	42.00^d^	38.33^d^	2.78	60.24
Seed treatment + seedling dipping	3.44	3.21	38.22^c^	35.66^cd^	2.94	61.54
Seedling dipping + Foliar spraying	3.24	3.00	36.11^c^	33.33^bc^	3.24	63.65
Seed treatment + Foliar spraying	2.89	2.87	32.11^b^	31.88^b^	3.40	65.87
Seed treatment + seedling dipping + Foliar spraying	1.53	1.28	17.00^a^	14.22^a^	3.43	67.82
Healthy (Untreated control)	8.80	7.21	97.00^f^	97.77^g^	1.90	26.54
SEd			0.95	1.05	0.01	0.39
CD			2.03	2.25	0.03	0.84

Means in columns followed by same letters are not significantly different (*p* < 0.05) according to DMRT.

## Discussion

Actinobacteria like *S. rochei* have long been a well-known biocontrol agent to inhibit the development of plant disease-causing microbes and boost crop immunity. Antibiosis, enzyme synthesis, enzyme suppressors, and other mechanisms of action have now been utilized by BCAs to manage plant fungal infections and signaling proteins (Sudha et al., 2017, 2019; Sayyed et al., 2019; Kiss et al., 2020). Antagonistic plant–microbe interactions might improve yield by 20% and decrease reliance on chemical pesticides and fertilizers by 20%. Only a few studies have addressed the microbial molecule structural diversity and functionality. However, there has been limited research on the compounds’ natural functions, which must be extended (Kalam et al., 2020; Moumbock et al., 2021). Hence, this study focuses on grain mold pathogens of sorghum and their management by studying the mechanism of inhibitory by volatiles secreted by *S. rochei*, which will eventually improve crop growth.

The results of the present study showed the antagonistic effect of *S. rochei* on the grain mold pathogens of sorghum, such as *F. moniliforme* and *C. lunata*, under laboratory conditions using the dual plate method. The mycelia of both the pathogens were malformed and distorted by the presence of *S. rochei*, so the results also demonstrated that it might have antifungal activity. Such results are in line with those of Liu et al. (2019) and Basu et al. (2021). They confirmed that *Streptomyces* spp. inhibited the mycelial growth of *Rhizoctonia solani* and *Sclerotinia sclerotiorum*, which are the causal agents of stem rot in the sunflower plant. *S. rochei* is broadly reported for the secretion of antimicrobial compounds used therapeutically. About 22% of the actinobacterial colonies isolated from the global environment exhibited inhibitory activity against pathogens, particularly against fungal strains (Das et al., 2018). Likewise, *S. rochei* isolated from the marine environment from mining sediment was a rich source of antimicrobial compounds (Tenebro et al., 2021). The results also coincide with the findings of Abdelmoteleb and González-Mendoza (2020). *Streptomyces netropsis* isolated from rhizospheric soil from *Larrea tridentata* exerted antifungal action more than *Macrophomina phaseolina*, *F. oxysporum*, *F. solani*, *F. equiseti*, *Botrytis cinerea*, *Alternaria alternata*, and *C. gloeosporioides*, with percent inhibition values ranging from 55.02 to 77.27%.

*Streptomyces rochei* ACTA1551, isolated from the rhizosphere of *Pinus brutia*, was also found to be capable of producing antifungal compounds (metabolites) and protecting tomato plants from *Fusarium oxysporum*. In this study, the pathogen hyphal filament was reduced in size with distortion and lysis of hyphae, as revealed in compound microscopic studies. This could be because of the emission of volatile and non-volatile compounds from *S. rochei* strains. Kim and Song (2016); Lyu et al. (2017), and Kim et al. (2019) reported that suitable results were obtained from this actinomycete fungus (*Streptomyces* spp.) against phytopathogens in the laboratory. Another supporting evidence was that the antagonist *S. rochei* was able to produce secondary metabolites that could penetrate and destroy the hyphae and cause lysis of *S. sclerotiorum* and *Pythium* sp. (Arora et al., 2021; Gebily et al., 2021).

### Volatile organic compound on tripartite bioassay

If the compounds secreted by the antagonist were diffused into the medium, it would reduce the growth of the pathogens. Our study also proved that the antagonist *S. rochei* could strongly inhibit the pathogens. The tripartite plate assays resulted in the highest inhibition percentages. Hence, the antimicrobial nature of the antagonist *S. rochei* might be accredited to organic molecules that are unstably secreted by *S. rochei*. Similarly, Wu et al. (2016, 2018) reported that mVOCs secreted by *Streptomyces* spp. resulted in abnormal growth characteristics in *S. sclerotiorum*. Spore germination by *F. moniliforme* was controlled by butane and dimethyl disulfide production through *Streptomyces* sp. Gotor-Vila et al. (2017) found that variation in susceptibility or resistance to VOCs will differ for pathogen levels. Furthermore, antibiosis is an important mechanism used by *Streptomyces* spp. to manage S. *sclerotiorum* sclerotia and *Aspergillus* contamination (Smolinska and Kowalska, 2018; Lyu et al., 2020).

Volatile organic compounds represent a number of untapped classes of metabolites, and further novel work is essential to thoroughly understand the ecological roles of these compounds and their role in disease management (Olanrewaju and Babalola, 2019). mVOCs from *S. rochei* were profiled with antifungal activities and proved through screening results. The extracted metabolites from *S. rochei* showed complete inhibition of pathogens at 50 and 75 μl dilutions the findings of Human et al. (2016) reported the antifungal activity of *Streptomyces* spp. This could be evidenced by the production of fungichromin, a polyene antifungal compound detected in extracts of infrutescene of Protea. Dewi et al. (2017) also proved the antifungal activity of metabolites of *Streptomyces* spp. against *Fusarium oxysporum* f. sp. *Cubense*. A number of strains in Streptomyces spp. have antifungal potential for *Magnaporthe oryzae* (*Pyricularia oryzae*), a fungus causing rice blast (Law et al., 2017). VOCs are emitted in different ways by various species. Other strains of *Streptomyces* sp. are being profiled in self-regulating isolates, and the interface suggests the discharge of certain new microbe-specific molecules. Various mVOCs exert antimicrobial activity against different pathogens to varying degrees. *F. moniliforme* emits unique volatile compounds responsible for virulence and pathogenesis. Other compound classes include an organic acid (acetic acid), two aromatic compounds (benzaldehyde and styrene), a monoterpene (beta-phellandrene), and a ketone (acetone). Compounds like oxalic acid co-butyl ester, succinaldehyde, aminocyanoacetic acid, allantoic acid, ifosfamide, tributylamine, and pyruvaldehyde were detected. However, during the interaction of *S. rochei* with *F. moniliforme*, we profiled acetic acid as the only compound. 5-Hydroxymethyl-2-furancarboxaldehyde compounds produced by *Streptomyces* JBS5-6 species inhibit the mycelial growth of Panama wilt pathogen (Zhou et al., 2017; Jing et al., 2020). Hence, our results revealed that disulfide dimethyl identified from *S. rochei* might be the compound responsible for the mycelial inhibition of *F. moniliforme*. These results indicate that although dimethyl disulfide and dimethyl trisulfide play a crucial function in inhibiting fungal growth, the mixture of volatiles affects the overall effect. The growth of mycelium, sporulation, or germination of conidia of *P. italicum* has been found to be affected by dimethyl disulfide released from *Streptomyces* sp. These findings suggest that VOCs from *S. rochei* have the ability to manage grain mold pathogens of Sorghum through fumigant action. In this study, terpenes were produced, which could have a synergistic effect and hence a stronger inhibitory effect than in previous studies. These results support the idea that a combination of sulfides with terpenes inhibits the growth of *F. culmorum* and induces a change in pigment production by *S. griseus* (Garcia et al., 2019). Non-dissociated acetic acid promotes lipid solubility, allowing increased fatty acid accretion in cellular membranes or other cell wall portions. However, being a weak acid, acetic acid can impede glucose absorption, resulting in cell death of pathogens (National Cancer Institute) (Chaves et al., 2021). Moreover, signaling during the disease development plays an important role in studying these tripartite interactions. Beccari et al. (2011) found that the mode of infection and interaction with the plant of root rot and Fusarium rots followed different pathways. These tripartite interaction also increased the growth attributes in plants by mycorrhizal and nitrogen-fixing symbiosis and raised their ability to induce plant growth (Schrey et al., 2012). Likewise, in this study, the pathways like sesquiterpenoid and alkane biosynthetic pathways would play a significant role in growth promotion and inhibition.

Zhang et al. (2017) discovered that α-phellandrene prevents the fungal development of *Penicillium cyclopium* by reducing the reliability of the cell membrane, resulting in the expulsion of biological molecules and potassium ions, a high lipid content, and changes in extracellular pH and membrane permeability. Similarly, in our study, α-phellandrene might be one of the compounds emitted by *S. rochei* during the interaction. It may act as a biofungicide to suppress grain mold pathogens in sorghum.

### Seed bacterization plant growth promotion

Biopriming with PGPR enhanced the seed growth other than through biological control (Almaghrabi et al., 2014). This result coincides with our investigations; the colonization of *S. rochei* on the root and shoot was higher than control. Similarly, Kunova et al. (2016) found that *S. anulatus* increased the radical growth of cultivated rocket by approximately 46.83 mm compared to the control (15.52 mm). These strains frequently produce IAA, stimulating cell elongation and root growth. Studies on growth promotion in rice by the strain *Streptomyces* VSMGT1014 showed seed germination improved root growth, shoot length, and fresh and dry weight of the vegetative parts compared to control (Khan et al., 2021).

The present study revealed that mVOCs from Actinobacteria promote plant growth. Olanrewaju and Babalola (2019) found that a few species of *Streptomyces*, including *S. anulatus* S37, *S. matansis*, *S. pulcher*, *S. vinaceus*, and a few others, also have plant growth promoting properties. Other compounds from the relationship are comprehensively addressed in the sections beneath. *Streptomyces* sp. have shown several PGPR characteristics such as IAA production, P solubilization, siderophores, and chitinase (Tamreihao et al., 2016), and these PGPR characteristics could be the reason for the growth promotion in roots of sorghum seedlings.

The research findings suggested that VOCs produced by *S. rochei* differ in their chemical nature and exhibit antimicrobial action and growth promotion properties. Also, these biomolecules are formed *in situ* in nature in the appropriate proportions and could be used as effective plant growth supporters and biological control mediators for sustainable crop production. As a result, VOC profiling can explore new compounds and metabolic pathways essential in antifungal action against grain mold pathogens of sorghum and plant growth promotion activity in sorghum seedlings. We identified a few VOCs with antifungal action against grain mold pathogens.

## Data availability statement

The original contributions presented in this study are included in the article/Supplementary material, further inquiries can be directed to the corresponding author/s.

## Author contributions

AS and DD conceived the study. SA performed the experiments. DD, AM, and TS coordinated the experiments on volatile metabolite, GC-MS, and analysis and interpretation of results. PP and RS contributed to conceptualization and writing – review and editing. ON and MA contributed to writing – review and editing and fund acquisition. All authors have read and approved the manuscript before submission.

## Supplementary material

Supplementary Figure 1 Molecular identification of sorghum grain mold pathogens using specific primers.

Supplementary Figure 2 Molecular phylogeny based on 16S rRNA sequences of Streptomyces isolates.

## Abbreviations

mVOCs: microbial volatile organic compounds
NIST: National Institute of Standards and Technology
PCA: principal component analysis
PC: principal component
PC1: principal component 1
PC2: principal component 2
PDA: potato dextrose agar
PGPR: plant growth-promoting rhizobacteria
VOCs: volatile organic compounds.
