# Supplementary figures and images for: Unraveling the tripartite interaction of volatile compounds of Streptomyces rochei with grain mold pathogens infecting sorghum
Source: Front Microbiol. 2022 Jul 28;13:923360. doi: 10.3389/fmicb.2022.923360 (PMC9366667; doi:10.3389/fmicb.2022.923360)

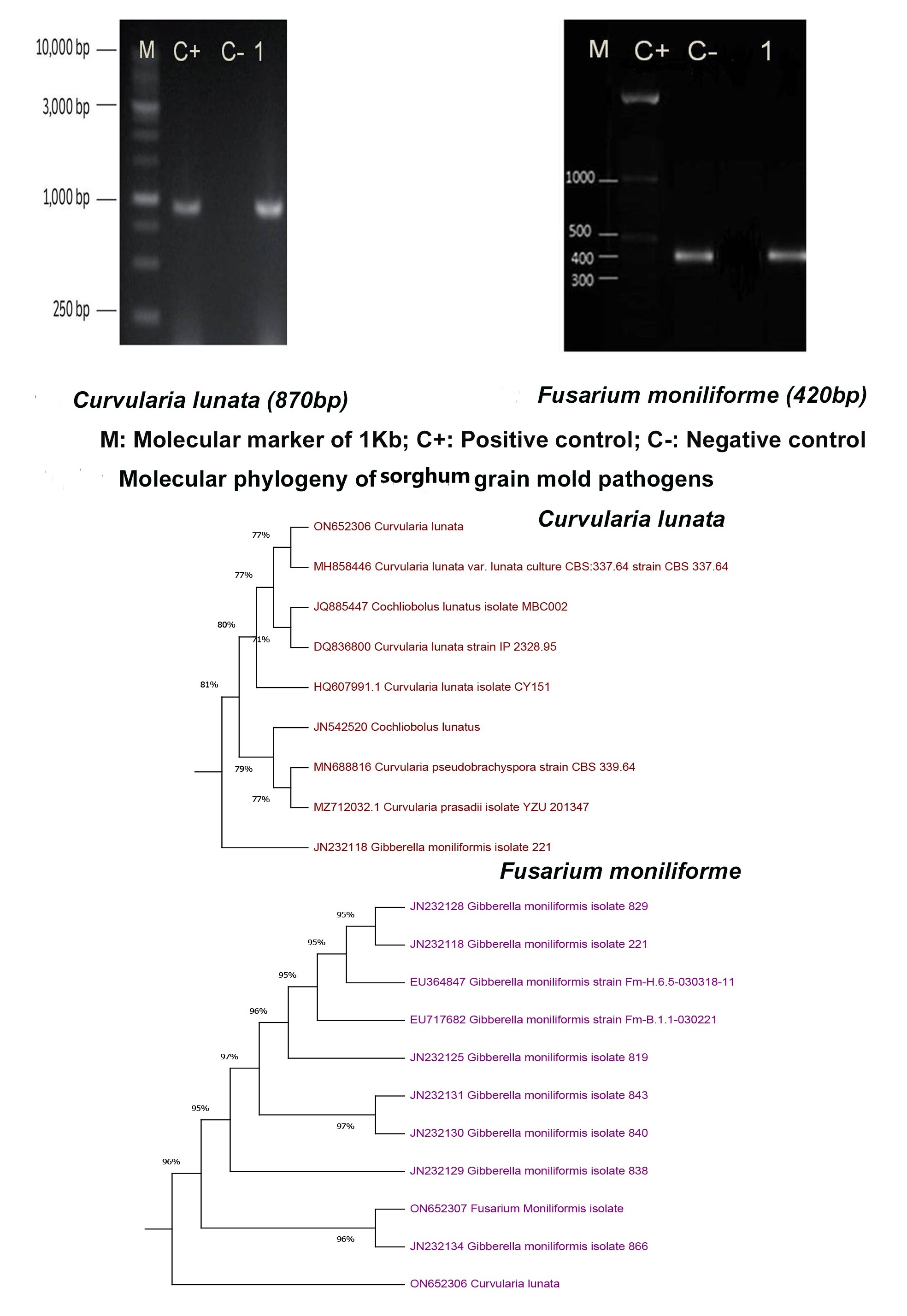

Supplement: Supplementary Figure 1 — Molecular identification of sorghum grain mold pathogens using specific primers. [file Image_1.JPEG]

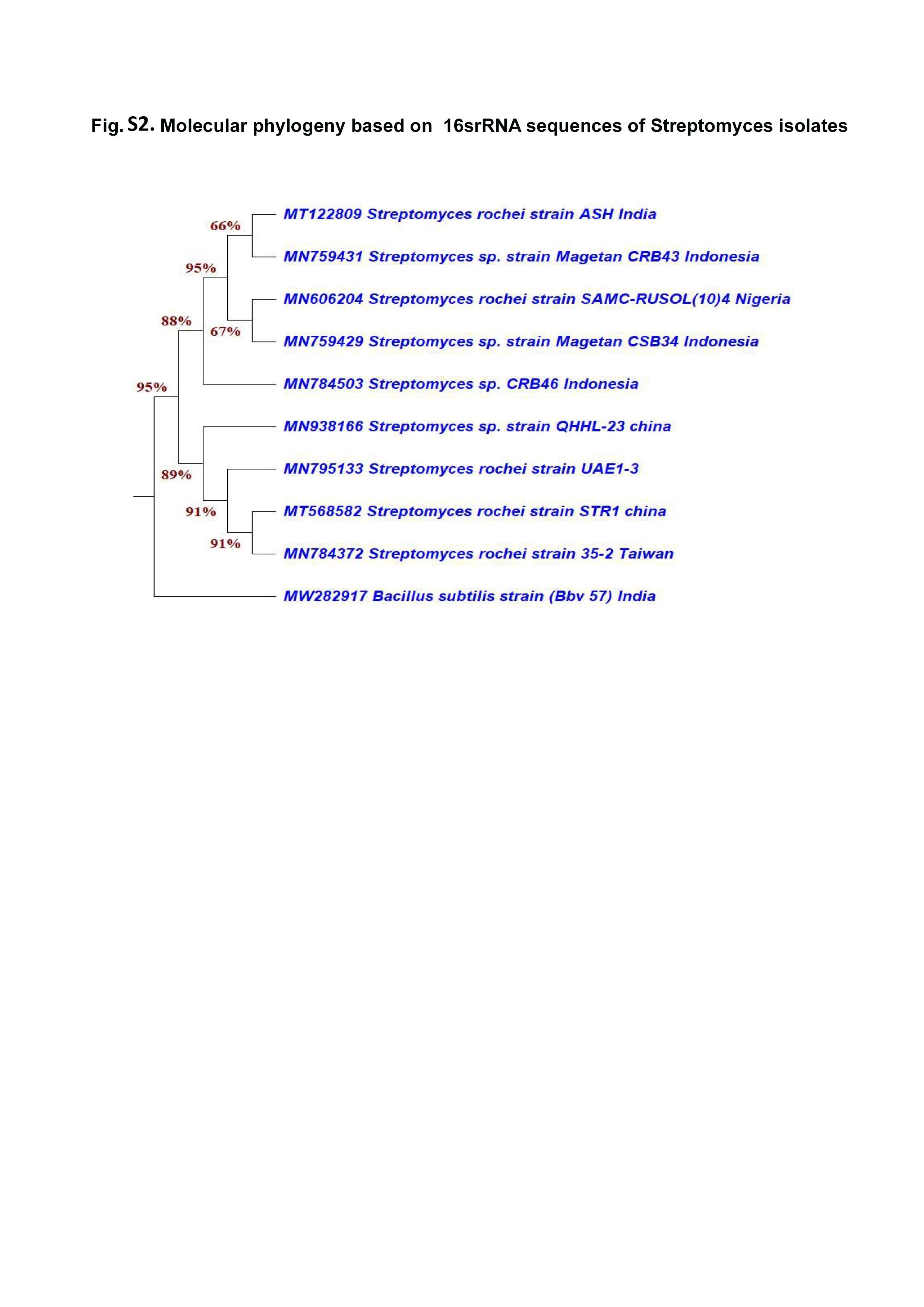

Supplement: Supplementary Figure 2 — Molecular phylogeny based on 16S rRNA sequences of Streptomyces isolates. [file Image_2.JPEG]
